# High-level C-X-C chemokine receptor type 4 expression correlates with brain-specific metastasis following complete resection of non-small cell lung cancer

**DOI:** 10.3892/ol.2014.1979

**Published:** 2014-03-18

**Authors:** LUNQING WANG, ZHOU WANG, XIANGYAN LIU, FANYING LIU

**Affiliations:** 1 Department of Thoracic Surgery, Provincial Hospital Affiliated to Shandong Univeristy, Jinan, Shandong 250021, P.R. China; 2 Department of Thoracic Surgery, Qingdao Municipal Hospital, Qingdao, Shandong 266071, P.R. China

**Keywords:** C-X-C chemokine receptor type 4, non-small cell lung cancer, metastases, brain

## Abstract

Brain-specific metastasis is one of the primary causes of recurrence following complete resection of non-small cell lung cancer (NSCLC) and the underlying mechanism remains unclear. The present study was designed to investigate the correlation between C-X-C chemokine receptor type 4 (CXCR4) expression and brain-specific metastasis of NSCLC. Lung cancer tissues from 105 patients who underwent complete tumor resection between January 1998 and June 2008 (sample group, 34 with brain metastasis during the follow-up period; control group 1, 34 without metastasis during the follow-up period; and control group 2, 37 with other organ metastasis, excluding brain metastasis, during the follow-up period) were examined by immunohistochemistry to detect the expression of CXCR4 protein. The differences in CXCR4 expression were compared using McNemar’s χ^2^ test. Estimation of survival was calculated with the Kaplan-Meier method and the statistical differences were analyzed with the log-rank test. Overexpression of CXCR4 protein was observed in 31 (91.2%) NSCLC patients with brain metastasis, which was greater than that observed in the NSCLC patients with other organ metastases (73.0%; P=0.048) and without metastases (14.7%; P<0.001). CXCR4 protein was highly overexpressed in patients with brain-specific metastasis, which indicated that high-level CXCR4 expression correlates with brain-specific metastasis of NSCLC.

## Introduction

Surgery is the preferred treatment for non-small cell lung cancer (NSCLC). However, without satisfactory surgical results, the overall five-year survival rate is ~30–40%. The majority of patients succumb following surgery due to tumor metastasis and recurrence. The brain metastases-associated clinicopathological features and molecular biological markers are clinically significant as they may determine a more accurate prognosis for the patient, allowing postoperative adjuvant therapies to be targeted. However, there is currently no effective method for identifying high-risk NSCLC patients with brain-specific metastasis.

Brain-specific metastasis is one of the primary causes of recurrence following complete resection of NSCLC, and the underlying mechanism remains unclear. The present study was designed to investigate the correlation between C-X-C chemokine receptor type 4 (CXCR4) expression and brain-specific metastasis of NSCLC.

The homing theory states that tumor cell metastasize to specific organs, due to an organ-specific capacity to arrest or attract specific types of cancer cells via chemotaxis (1). Therefore, the spreading of certain tumors is considered to be selective, rather than random This hypothesis is further affirmed by the observations of organ-specific metastasis in certain cancer types, such as prostate cancer, where the tumor cells are more likely to metastasize to bone (2). Previous studies have identified that CXCR4 and its ligand, CXCL12, are associated with organ-specific metastasis (2–4). Our previous preliminary study demonstrated that CXCR4 overexpression in tumor tissue is correlated with NSCLC at the homeochronous and heterochronic phases of solitary brain metastasis (5).

Based on previous studies, the present study retrospectively investigated NSCLC patients with brain metastasis following complete resection. Immunohistochemical methods were used to detect the expression of CXCR4 within tumor tissues. Through matched-pair analysis the correlation between CXCR4 overexpression and brain metastasis of postoperative NSCLC patients was investigated. The function of CXCR4 in brain-specific metastasis of NSCLC, according to the controlled analysis of patients with brain-specific metastasis and patients with other organ metastases, was also examined.

## Patients and methods

### Patients

Between January 1998 and June 2008, 5,117 patients underwent surgical resection of NSCLC at the Qingdao Municipal Hospital (Qingdao, China). A total of 105 (2.1%) patients who underwent complete tumor resection were retrospectively reviewed. The patients in the present study did not receive any neoadjuvant therapy prior to surgery. This study was approved by the ethics committee of Qingdao Municipal Hospital (Qingdao, China). Patients provided written informed consent.

The sample group included 34 NSCLC patients with brain-specific metastasis during the follow-up period, which consisted of 22 males and 12 females, with a median age of 56 years (range, 37–75 years). Histological analysis revealed that there were 14 squamous cell carcinomas, 17 adenocarcinomas and three large cell lung cancers. According to the 2009 International Association for the Study of Lung Cancer tumor, node, metastasis (TNM) classification, seven patients were classified as Stage I, 11 patients were classified as Stage II and 16 patients were classified as Stage III. A total of 30 patients received 4–6 cycles of cisplatin-based chemotherapy (Table I).

### Follow-up and diagnosis of metastases

A complete patient follow-up was performed on a regular basis. Every 3–6 months, the patients received comprehensive medical examinations as follows: Brain and thorax X-ray computed tomography (CT) scans; upper abdomen enhanced CT scans; and/or liver, gallbladder, pancreas, spleen, kidney and adrenal B-mode ultrasound; electroconvulsive therapy scans in the patients presenting with bone pain; and whole body PET-CT in certain patients. The newly identified lesions in the brain and other organs were diagnosed as metastases after eliminating the possibility of benign lesions. The pathological investigation following surgical resection confirmed brain-specific metastasis in 16 patients and the remaining patients were clinically diagnosed with brain-specific metastasis.

### Research design and statistical analysis

A total of 34 eligible NSCLC patients without metastases during the follow-up period served as control group 1 (Table I) and 37 NSCLC patients with other organ metastases, excluding brain-specific metastasis, during the follow-up period served as control group 2 (Table II). All of these patients were screened in the following order: TNM stage, histological tumor type, degree of tumor differentiation, gender, surgical pattern, adjuvant therapy post-surgery and age. When these clinicopathological characteristics did not exactly match with the experimental group, the maximum extent of consistency principle was followed.

Pearson’s χ^2^ test was used to evaluate differences in CXCR4 expression between the patients with brain-specific metastasis and the patients without hematogenous metastases; this enabled examination of the correlation between CXCR4 expression and postoperative brain-specific metastasis of NSCLC. The patients who received complete tumor resection at the same period as the other two groups and exhibited other organ metastases during the follow-up period were selected for control group 2. The χ^2^ test was used to evaluate differences in CXCR4 expression between brain-specific metastasis patients and other organ metastases patients in order to further examine the role of CXCR4 in postoperative brain-specific metastasis of NSCLC.

The clinical and pathological data were compiled into an SPSS 15.0 database (SPSS, Inc., Chicago, IL, USA). An estimation of survival rate was calculated using the Kaplan-Meier method and statistical differences were analyzed with the log-rank test. P<0.05 was considered to indicate a statistically significant difference.

### Techniques

Immunohistochemistry (streptavidin-peroxidase [SP] method) was performed to detect the CXCR4 expression levels in every tissue specimen. The primary antibody employed was the mouse anti-human CXCR4 monoclonal antibody at 1:100 dilution (Abcam Ltd.). Secondary processing of the tissue samples was performed with an SP kit and a universal secondary antibody kit according to the manufacturer’s instructions (Beijing Zhongshan Golden Bridge Biotechnology Co., Ltd., Beijing, China). Briefly, following incubation overnight with the primary antibody, the secondary biotinylated antibody and subsequent avidin-biotin complex reagent were incubated for 30 min, respectively. Staining was visualized using diaminobenzidine.

**Figure 1:**
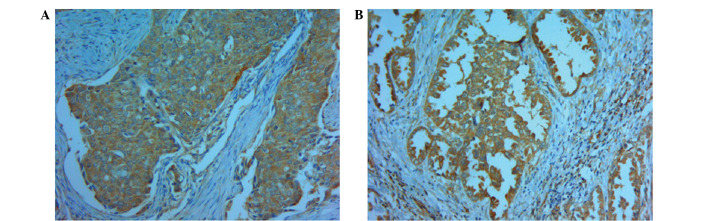
C-X-C chemokine receptor type 4 expression in non-small cell lung cancer. (A) Lung squamous cell carcinoma. (B) Lung adenocarcinoma. (Magnification, ×200). Streptavidin peroxidase immunohistochemical staining was used for visualisation.

Paraffin-embedded specimens of the surgically removed tumor tissue were collected and all of the stained sections were examined by two independent experienced pathologists who had been double blinded to the clinical data. The immunohistochemical score was calculated by combining the proportion score (percentage of positive stained cells) with the staining intensity score. The proportion score ranged from 0 to 4, as follows: 0 (no staining), 1 (1–24%), 2 (25–49%), 3 (50–74%), 4 (>75%). The staining intensity was scored as follows: 0 (negative), 1 (weak), 2 (moderate) and 3 (strong). The proportion score and staining intensities score were subsequently multiplied to generate the total IHS score for each case. The total score ranging 4–12 was considered to be positive expression. The tissue specimens were scored according to a combination of the intensity and the proportion of positive-stained tumor cells, as follows: i) The number of positive tumor cells was determined by assesment of l0 high-power fields of dense tumor cell areas, which were randomly selected for counting. A minimum of 600 tumor cells were counted and observed and the proportion of the positive-stained tumor cells was evaluated according to the following scale: 0=0–5%; 1=6–10%; 2=11–50%; and 3≥50%. ii) The staining intensity was evaluated according to the following scale: 0=No reactivity; 1=low; 2=moderate; and 3=strong. A total score of 3–6 was considered to indicate positive staining of CXCR4 (Fig. 1) (6).

## Results

### The difference in CXCR4 protein expression between patients with brain-specific metastasis and the control groups

In the group of patients with brain-specific metastasis, CXCR4 overexpression was observed in 31 samples of NSCLC tissue (91.2%), whereas of the 34 patients without metastases, CXCR4 overexpression was observed in five samples of NSCLC tissue (14.7%), which was significantly lower compared with that of patients with brain-specific metastasis according to the χ^2^ tests (P<0.001; Table III).

In the group of patients with other organ metastases, CXCR4 overexpression was observed in 27 samples of NSCLC tissue (73.0%), which was significantly higher compared with that of patients without metastases (14.7%; P<0.001; Table III).

The CXCR4 expression levels of the brain-specific metastasis group and other organ metastases group (control group 2) were compared. According to the χ^2^ test (Pearson method) the brain-specific metastasis patients exhibited a higher expression of CXCR4 (Table III), which was identified to be a statistically significant difference (P=0.048).

### Analysis of survival rate among NSCLC patients with and without metastases

**Figure 2:**
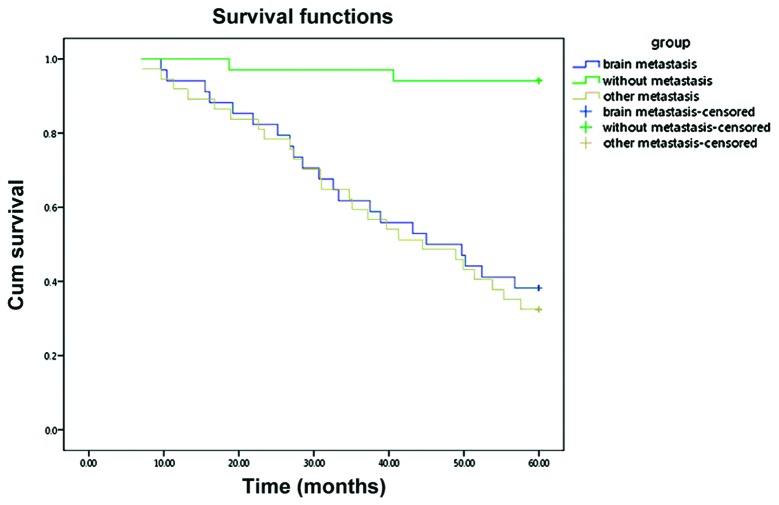
Kaplan-Meier curve of five-year cumulative survival rate.

All patients with lung cancer were closely followed-up, with a mean follow-up time of 59.6 months (range, 7–116 months). The three- and five-year cumulative survival rates of 34 patients with brain-specific metastasis were 61.8 and 38.2%, respectively and the three- and five-year cumulative survival rates of patients with other organ metastases were 59.5 and 32.4%, respectively. The log-rank test demonstrated that there were no statistically significant differences identified between the survival rates of the two groups (P>0.05; Fig. 2). The three- and five-year cumulative survival rates of the 34 patients without metastases were 97.1 and 94.1%, respectively.

## Discussion

Clinicopathological feature analyses have revealed that, for NSCLC patients presenting with brain-specific metastasis alone, metastatic lesion resection or other adjuvant therapies may improve quality of life and prolong survival time (7–9). Therefore, identifying patients with a high risk of brain-specific metastasis following lung cancer resection is clinically significant for predicting the prognosis and selecting the most appropriate adjuvant therapy.

Currently, the mechanism(s) that promote brain-specific metastasis have not been clearly elucidated. When NSCLC attacks the pulmonary vein, tumor cells are able to directly enter the blood circulation without pulmonary capillary bed involvement. Hematogenous spread may lead to multiple organ metastases, with the brain being the most common metastatic site clinically. Further studies are required to determine why NSCLC cells remain in the brain by hematogenous metastases and the underlying mechanisms regarding the specific affinity of NSCLC cells to the brain.

In recent years, the homing theory has been proposed as a result of investigations into tumor metastases to specific organs. The theory states that different organs chemotactically capture or attract particular types of tumor cells, which is termed homing and results in metastasis to specific organs (1). In 2001, Müller *et al* (3) proposed that tumor cells metastasize by a specific combination of chemotactic factors (chemokines) and receptors (chemokine receptor) to specific organs. It was identified that breast cancer cells express CXCR4 highly and that the ligand of CXCR4, CXCL12, is primarily expressed in the lungs, liver and bone marrow. It is also these same organs that the majority of breast cancer cells often metastasize to, providing strong correlational evidence in support of the homing theory. Clinically, NSCLC most commonly metastasizes to the brain. Whether this is also due to an interaction between chemotactic factors and their receptors requires further investigation.

In our previous study, the correlation of CXCR4 and solitary brain-specific metastasis at the homeochronous and heterochronic phases was examined. The preliminary results demonstrated that CXCR4 expression levels in the tumor tissue of NSCLC patients with brain-specific metastasis is higher compared with NSCLC patients without distant metastases (5). This indicated that CXCR4 may be associated with brain-specific metastasis in NSCLC. In order to further examine the correlation between CXCR4 overexpression and brain-specific metastasis following NSCLC surgical resection and to assess whether it is associated with brain-specific metastasis, the present study examined more cases. In addition, a comparison between patients with brain-specific metastasis and patients with other organ metastases was performed.

In the group of patients with brain-specific metastasis, CXCR4 overexpression was observed in 31 of the patients with brain-specific metastasis and, of the 34 patients without metastases, CXCR4 overexpression was observed in only five patients, which was a statistically significant difference. In the group of patients exhibiting other organ metastases, CXCR4 over-expression in tumor tissue was also higher compared with the patients without metastases. The present study indicates that CXCR4 overexpression in NSCLC may be correlated with postoperative hematogenous metastases. Further analysis by comparing patients exhibiting brain-specific metastasis and other organ metastases revealed that CXCR4 overexpression is higher in patients with brain-specific metastasis compared with patients exhibiting other organ metastases (P=0.048; Table III). It was also demonstrated that a chemotaxis function may mediate the homing of CXCR4-overexpressing NSCLC cells to the brain, where the ligand CXCL12 is overexpressed.

Adopting statistical methods of matching comparison reduced the experimental bias and enhanced the objectivity of the present study. However, as a retrospective study, several limitations remain, including: i) Following surgery, the adjuvant chemotherapy scheme and medication-use time were not tightly controlled between the patients; ii) the majority of organ metastases patients were clinically diagnosed; iii) the number of cases involved in this single-center study was limited; iv) due to ethical considerations, it was not possible to obtain normal brain tissue for the detection of normal CXCL12 expression levels. However, the fact that CXCL12 is constitutively expressed in the developing and mature central nervous system (10,11) may support the results of the present study indirectly. Previous studies have demonstrated that CXCL12 expression was normally controlled at a relatively low level (12–17). Under certain pathological situations, including HIV 1-associated dementia, brain tumor, ischemia and neuroinflammation, CXCL12 expression may be briefly upregulated. Astrocytes and vascular endothelial cells in the parenchyma have been proposed as two primary cell sources for inducible CXCL12, and hypoxia-inducible factor-1 (18) may regulate CXCL12 gene expression in endothelial cells, resulting in the selective expression of CXCL12. Furthermore, interleukin-1β (16) induces CXCL12 in astrocytes by extracellular signal-regulated kinase and phosphatidylinositol-4,5-bisphosphate 3-kinase signaling pathways. In the present study, although the majority of organ metastases occurred within three years postoperatively and all of the patients in the control groups had regular follow-up, no brain-specific metastasis was identified; however, the possibility that brain-specific metastasis may have occurred subsequently may not be ruled out. These problems should be addressed in future studies.

In conclusion, brain-specific metastasis is one of the primary reasons for recurrence following complete resection of NSCLC. At present, it is only possible to assess the occurrence of brain-specific metastasis according to clinicopathological features, which are considered to lack sensitivity. The present study demonstrated that CXCR4 overexpression in patients with brain-specific metastasis was higher when compared with the control group patients, indicating that the CXCL12/CXCR4 signaling axis may be involved in the brain-specific metastasis processes of NSCLC. Therefore, further studies are required to examine whether CXCR4 may be a molecular marker in predicting brain-specific metastasis associated with NSCLC.

## Figures and Tables

**Table I:** Clinicopathological features of patients with brain-specific metastasis and patients in control group 1.

Clinicopathological feature	Brain metastasis (no. of cases)	Control group 1 (no. of cases)
Age (years)		
<40	4	3
40–50	6	5
50–60	15	15
60–70	7	8
>70	2	3
Gender		
Male/Female	22/12	21/13
Surgical pattern		
R. upper lobe	8	9
R. lower lobe	7	8
R. middle lobe	2	1
R. middle and lower lobes	3	2
R. whole lung	1	1
L. upper lobe	6	6
L. lower lobe	5	6
L. whole lung	2	1
Pathological type		
Adenocarcinoma	17	18
Squamous cell carcinoma	14	14
Large cell carcinoma	3	2
Degree of differentiation		
Well differentiated	13	13
Poorly differentiated	18	19
Undifferentiated	3	2
T status		
T1/T2/T3/T4	5/16/8/5	6/17/8/3
N status		
N0/N1/N2	7/15/12	8/17/9
Post-operative adjuvant chemotherapy		
Yes/No	30/4	31/3
Diagnosis of brain metastases		
Pathology/Imaging	16/18	0
Transfer time from surgery		
<6 months	2	
6–12 months	6	
12–24 months	12	
24–36 months	9	
>36 months	5	

R, right; L, left.

**Table II:** Clinicopathological features of patients with other organ metastases in control group 2.

	Location of metastases^a^				
	
Clinicopathological features	Lung and pleura (n=14)	Liver (n=10)	Bone (n=6)	Adrenal glands (n=5)	Other (n=2)
Surgical pattern					
Lobe/Whole lung	12/2	9/1	6/0	5/0	1/1
Pathological type					
Adenocarcinoma	7	5	2	1	0
Squamous cell carcinoma	5	5	4	3	2
Large cell carcinoma	2	0	0	1	0
Degree of defferentiation					
Well differentiated	5	6	3	2	2
Poorly differentiated	7	3	3	3	-
Undifferentiated	2	1	0	0	0
TNM stage					
I	2	1	0	1	0
II	5	4	2	1	2
III	7	5	4	3	0
Diagnosis of metastases					
Pathology/Imaging	6/8	1/9	1/5	3/2	2/0

a The location of metastases at first diagnosis.

TNM, tumor, node, metastasis.

**Table III:** Comparison of CXCR4 expression in non-small cell lung cancer patients.

	Expression of CXCR4		
	
Location of metastases	Positive	Negative	Total
Brain	31	3	34
Other organ	27	10	37
No metastases	5	29	34

CXCR4, C-X-C chemokine receptor 4.
